# 3D-Printable Granular Hydrogel Composed of Hyaluronic Acid-Chitosan Hybrid Polyelectrolyte Complex Microgels

**DOI:** 10.1021/acs.biomac.5c00228

**Published:** 2025-05-22

**Authors:** Armin Amirsadeghi, Shahriar Mahdavi, Paula Jager, Marleen Kamperman, Julien Es Sayed

**Affiliations:** † Zernike Institute for Advanced Materials, 332143 University of Groningen, Nijenborgh 3, Groningen 9747 AG, The Netherlands; ‡ Biotechnology Centre, 3647 The Silesian University of Technology, B. Krzywoustego 8, Gliwice 44-100, Poland; § Department of Biomedical Engineering, University of Groningen, University Medical Center Groningen, A. Deusinglaan 1, Groningen AV 9713, The Netherlands

## Abstract

When it comes to 3D printing of hydrogels, optimizing rheological properties is crucial to ensure (i) a smooth flow of the ink through the nozzle during printing and (ii) structural integrity postprinting. Granular hydrogels offer excellent printability due to their intrinsic yield-stress properties; however, they typically lack postprinting integrity in aqueous environments. To address this limitation, a novel approach is proposed to integrate the yield-stress behavior of granular hydrogels with polyelectrolyte complexes, which exhibit tunable mechanical properties and structural integrity in aqueous media. In this approach, hybrid microgels composed of chemically co-cross-linked hyaluronic acid–chitosan polyelectrolytes are prepared in a high-salt medium to shield electrostatic interactions and then jammed to form a printable granular hydrogel. By reducing the salt concentration below the critical threshold for electrostatic association, intra- and interparticle electrostatic interactions are activated, causing both the microgels and the granular hydrogel to shrink. This process yields a hydrogel with tunable stiffness, packing density, and dimensions. Overall, this strategy paves the way for the design of 3D hydrogel constructs with dynamic properties.

## Graphical abstract

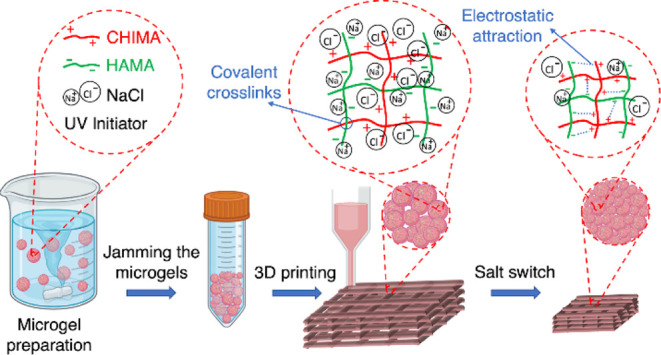

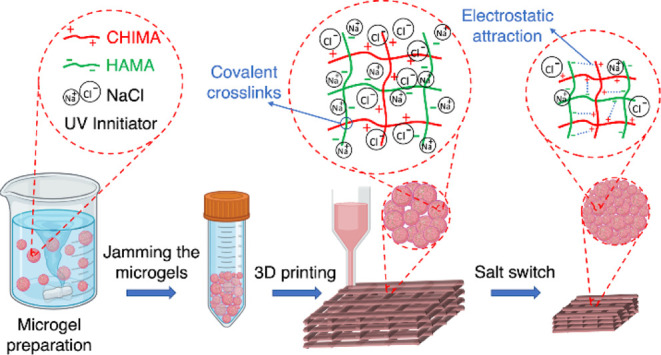

## Introduction

Granular hydrogels are a class of hydrogels made of closely packed, or jammed, microgel particles that have shown great promise as advanced inks for 3D (bio)­printing.^1,2^ Within this framework, their big advantage lies in the fact that they exhibit intrinsically favorable yield-stress properties. It allows them to behave as a free-standing elastic solid at rest but also to spontaneously flow when a stress overpassing the so-called yield-stress is exerted.^3–5^ At the particle scale, the flow is initiated only when the frictional energy to move the particles out of the “cage” in which they are entrapped by the neighboring ones is overcome. This process, which only relies on geometrical constraints, does not involve any specific chemical requirement on the particles. Moreover, granular hydrogels exhibit great versatility, as their particle size and chemical composition can be tailored to enhance the functionality of 3D-printed structures for tissue engineering.^1,6^ However, to fully harness their potential, strong interparticle interactions must be introduced to maintain structural integrity in aqueous environments.^7,8^ Up to now, several strategies have been investigated to mechanically reinforce granular hydrogels including the formation of interparticle (dynamic) covalent bonds,^9–11^ a percolating second network,^7,12–14^ metal–ligand interactions,^15,16^ or other supramolecular interactions (host–guest or electrostatics).^17,18^ Yet, while supramolecular interparticle interactions were shown to be efficient in enhancing the integrity of the printed structures, their intrinsic tunable strength of interactions has not been used to control the whole scaffold’s dynamic properties. In the present study, we envision that leveraging electrostatic interactions to tune both intra- and interparticle interactions will enable the development of structures with enhanced structural integrity but also tunable dimensions and mechanical properties.^19,20^

In this context, polyelectrolyte complexes (PECs) utilize electrostatic interactions between oppositely charged polyelectrolytes in aqueous media to develop hydrogel systems with tunable mechanical properties and swelling degree.^21–28^ For a given polyelectrolyte couple, the mechanics and swelling degree are dictated by the dynamics of the electrostatic associations that are directly dependent on the physicochemical conditions of the surrounding medium, of which pH (in the case of weak polyelectrolytes) and ionic strength are the most critical. As a general example, for a relatively low polyelectrolyte charge density or at relatively high salt concentration, PECs can behave as a free-flowing viscous liquid. Oppositely, at high polyelectrolyte charge density or low salt concentration, PECs can be obtained as self-standing elastic hydrogels. This continuum of behavior has already been advantageously used to propose novel 3D-printable hydrogel inks made of synthetic polymers (polystyrene-4-sulfonate, poly­(acrylic acid), poly­(diallyldimethylammonium chloride)), and/or natural polymers (hyaluronic acid, HA, chitosan, CHI, and gelatin) which were readily extrudable.^26,29–32^ For some of these systems, the ink immediately turned into a solid hydrogel scaffold upon printing in an aqueous bath containing a low concentration of salt. Consequently, scaffolds purely based on electrostatic interactions that exhibited satisfying shape retention over weeks when immersed in aqueous medium could be developed thanks to this so-called “salt switch”. A different approach was reported by Gong et al., who used polyelectrolyte complexation in a postprinting process. This resulted in 3D printed negatively charged HA-based hydrogel scaffolds which resolution could be enhanced by immersion in a bath containing positively charged CHI chains.^19^

Hence, we believe that the structural integrity and mechanical properties of granular hydrogels can be controlled by introducing polyelectrolyte complexation with a tunable strength depending on the environmental condition. More precisely, we propose a new design of microgels in which the polyelectrolyte complexation can be both triggered within a single particle, inducing a contraction of the particles, and in between particles, improving the structural integrity in an aqueous environment and possibly triggering the contraction of the whole structure. For this, negatively charged HA and positively charged CHI were used as polyelectrolytes. HA and CHI were first methacrylated, then co-cross-linked by UV-illumination within water drops in oil by an inverse emulsion process. A high enough salt concentration (1.5 M NaCl) was used to avoid early polyelectrolyte complexation during the microgel preparation process. The effect of the salt concentration of the medium on the size of the single microgels, on the yield-stress behavior, packing density, and structural integrity of the granular hydrogels was then investigated by combination of confocal laser scanning microscopy (CLSM) and rheology. Finally, the granular hydrogel was 3D-printed and the salt-responsive structural contraction was investigated.

## Experimental Section

### Materials

Chitosan (CHI), with a degree of deacetylation of 89% and an average molecular weight of 30 kg/mol, and sodium hyaluronate—referred to as hyaluronic acid—(HA), with an average molecular weight of 30–50 kg/mol, were both purchased from Glentham Life Sciences Co. (Corsham, United Kingdom). Light mineral oil, sodium chloride (NaCl), SPAN 80, TWEEN 20, methacrylic anhydride (MA), rhodamine B isothiocyanate (RITC), lithium phenyl-2,4,6-trimethylbenzoylphosphinate (>95%, LAP), 1-ethyl-3-(3-(dimethylamino)­propyl)­carbodiimide (>99%, EDC), and *N*-hydroxysuccinimide (>99%, NHS) were purchased from Sigma-Aldrich. BDP FL amine was purchased from Lumiprobe Corporation. All chemicals were used as received. Deionized (DI) water was produced by reverse osmosis (conductivity <10 μS/cm).

### BODIPY Labeled Hyaluronic Acid Methacrylate (HAMA) Synthesis

**1/Methacrylation of HA.** HA (1 g, 2.5 mmol of repeat units) was dissolved in 100 mL of a 0.25 M carbonate buffer aqueous solution (pH 9, 35.18% sodium carbonate, and 64.82% sodium bicarbonate) in a 500 mL round-bottom flask at room temperature. MA (2.0 mL, 13.5 mmol, 5.4 equiv) was added dropwise to the reaction medium. The pH of the medium was monitored to be 8 every hour for the first 6 h of the reaction by adding 1 M NaOH stock solution. The reaction was left to run at room temperature for 24 h. HAMA was further purified by dialysis (MWCO = 12–14 kDa, Spectra/Por 2, Spectrum Laboratories) for 3 days, changing the dialysis bath with deionized water twice a day to ensure complete removal of the carbonate salt, the unreacted MA. and the methacrylic acid molecules formed by hydrolysis of MA in water (followed by conductivity measurements). HAMA was finally obtained as a white, fluffy powder after freeze-drying. **2/Fluorescent labeling of HAMA.** HAMA (2.0 g), EDC (0.3 g, 1.56 mmol), and NHS (0.1 g, 0.46 mmol) were consecutively dissolved in 100 mL of aqueous 1×-PBS buffer (pH 7.2). Then, BDP FL amine (4.25 mg, 0.01 mmol) was directly dissolved in the reaction medium. The reaction mixture was left to run overnight at room temperature. Fluorescently labeled HAMA was further purified by dialysis (MWCO = 12–14 kDa, Spectra/Por 2, Spectrum Laboratories) for 3 days changing the dialysis bath with deionized water twice a day to ensure complete removal of residual BDP FL amine (followed by UV–visible spectroscopy), EDC, NHS, and urea side-product of the reaction (followed by conductivity measurements). BODIPY labeled HAMA (HAMA BDP) was finally obtained as a yellow fluffy powder after freeze-drying.

### Rhodamine B Labeled Chitosan Methacrylate (CHIMA) Synthesis

**1/Methacrylation of CHI.** CHI (1 g, 6.0 mmol of d-glucosamine) was dissolved in 80 mL of water, and the pH was adjusted to 5 using a 1 M HCl stock solution in a 500 mL round-bottom flask at room temperature. The temperature of the solution was adjusted to 40 °C and then MA (4.1 mL, 27.6 mmol, 4.6 equiv) was further added dropwise to the reaction medium. The reaction was left to run at 40 °C for 24 h. CHIMA was further purified by dialysis (MWCO = 12–14 kDa, Spectra/Por 2, Spectrum Laboratories) for 3 days to ensure complete removal of the unreacted MA and the methacrylic acid molecules formed by hydrolysis of MA in water. CHIMA was finally obtained as a white fluffy powder after freeze-drying. **2/Fluorescent labeling of CHIMA.** CHIMA (1 g) and RITC (0.01 g, 0.02 mmol) were consecutively dissolved in 50 mL of deionized water with a pH adjusted to 5 by the addition of 1 M HCl aqueous stock solution to ensure full solubility of CHIMA. The reaction was left to run overnight at room temperature. Fluorescently labeled CHIMA was further purified by dialysis (MWCO = 12–14 kDa, Spectra/Por 2, Spectrum Laboratories) for 5 days changing the dialysis bath with deionized water twice a day to ensure complete removal of residual RITC (followed by UV–visible spectroscopy). RITC labeled CHIMA (CHIMA RB) was finally obtained as a pink fluffy powder after freeze-drying.

### Hybrid HAMA-CHIMA Microgels Preparation

The hybrid HAMA-CHIMA microgels preparation protocol was adapted from a previously reported study.^33^ First, 5 wt % CHIMA (pH adjusted to 5) and HAMA stock solutions at a NaCl concentration of 1.5 M were prepared separately. Second, both stock solutions were mixed at a 1:1 volume ratio to obtain a homogeneous solution of 2.5 wt/v% HAMA and 2.5 wt/v% CHIMA. Third, LAP was directly dissolved in the mixture protected from external light at a concentration of 0.1 wt/v%. Fourth, 2 mL of the microgel precursor solution was emulsified on a magnetic stirrer in 6 mL of light mineral containing 2 wt % of SPAN 80 in a 20 mL glass scintillation vial at a stirring speed of 350 rpm using a 0.5 mm × 20 mm cylindrical magnetic stirring bar. After 10 min under stirring, the emulsion was illuminated under continuous stirring for 10 min using an LED strip (realUV LED Strip Lights from Waveform Lighting, λ = 390 nm, 117 LEDs, distance diodes-center of the vial = 7 cm, total power = 17 W, illumination density = 0.03 W/cm^2^) to trigger the polymerization of the methacrylate units and subsequent cross-linking of HAMA and CHIMA together. The detailed setup is reported in Figure S1. The microgels were further purified by cycles of centrifugation and redispersion (3 times in light mineral oil at 500 rcf, then 1 time in an aqueous solution at 1.5 M NaCl containing 1 wt % Tween 20 at 1500 rcf, and 10 times in a 1.5 M NaCl aqueous solution).

### Hybrid HAMA-CHIMA Bulk Hydrogel Preparation

The solution containing 2.5 wt/v % HAMA, 2.5 wt/v % CHIMA, 0.1 wt/v % LAP, and a NaCl concentration of 1.5 M was prepared as described before and then poured in a silicone cylindrical mold (diameter = 11 mm for the 1.5 M NaCl hydrogel and 13 mm for the 0.15 and 0.5 M NaCl hydrogels, height = 1 mm) and sealed with two glass slides. The solution was then illuminated under UV (similar setup as used for the microgels) for 1 min to obtain cross-linked HAMA-CHIMA hybrid homogeneous hydrogel.

### Hybrid HAMA-CHIMA Granular Hydrogel Preparation

The HAMA-CHIMA hybrid microgels were first dispersed in aqueous solutions containing 0.15, 0.5, or 1.5 M NaCl. They were then jammed by centrifugation at 4500 rcf for 20 min to ensure full removal of the excess water. Subsequently, the supernatant was removed, and centrifugation was repeated twice to ensure full removal of the excess water.

### 3D Printing of the Granular Hydrogel

3D printing was performed using a GeSiM BioScaffolder 5.3 instrument. The cartridge loaded with jammed microgels was placed on the 3D printer, and a 0.41 mm diameter plastic nozzle was attached to it. A square mesh design with an 8 × 8 mm, with 1.2 or 2 mm strand-to-strand distance (between the centers of two adjacent strands), was chosen. The pressure was set to 50 kPa to extrude the microgel ink. The printing speed was set at 2 mm/s, and the strand height was set equal to the width of the nozzle which was 0.410 mm.

The printability index (Pr) was determined from bright-field microscopy images of two-layer square-mesh scaffolds printed with a strand-to-strand distance of 1.2 mm. Images were captured immediately after printing, and the area (*A*) and perimeter (*P*) of individual pores were measured by using ImageJ software. The printability index was calculated using the following equation: $\mathrm{Pr}=\frac{P^{2}}{16A}$ , where *A* is the area and *P* is the perimeter of a single pore. For each formulation, three independent scaffolds were analyzed and six pores per scaffold were measured. The reported Pr values represent the average ± standard deviation across all measurements. Representative images used for this analysis are provided in Figure S14.

### Proton Nuclear Magnetic Resonance (^1^H NMR)

^1^H NMR spectra were acquired on an Agilent 400-MR 400 MHz spectrometer at 25 °C. Samples were dissolved in deuterated water and measured with a pulse width of 45 μs and recycle delay of 1 and 128 scans. Chemical shifts were determined from tetramethylsilane referenced to the residual isotopomer solvent signal (HOD).

### Rheology

An Anton Paar MCR302 rheometer (Anton Paar Co., The Netherlands) with a plate–plate geometry (top plate diameter of 10 or 25 mm) was used to perform small amplitude oscillatory shear rheology. An HR-2 rheometer (TA Instruments, United States) with an 8 mm plate–plate geometry was used to perform small amplitude oscillatory shear rheology on HAMA-CHIMA bulk hydrogels. The analyses were performed at a controlled temperature of 20 °C. The granular hydrogel was collected by using a spatula and loaded on the bottom plate of the rheometer. The gap was fixed at values between 300 and 1000 μm depending on the thickness of the hydrogels, and then a low-viscosity oil (Fluka silicon oil, viscosity 50 mPa·s) was added around the geometry to prevent solvent evaporation unless otherwise stated. After the normal force decreased stabilized to a constant value (between 0 and 1 N depending on the microgel concentration), a strain sweep (strain γ = 0.1 to 1000% and angular frequency ω = 1 rad/s) was performed to confirm the materials’ linear viscoelastic regime (LVE) and its yield-stress properties. The shear stress is calculated using the following formula: $\sigma =\gamma^{*}\sqrt{G{’}^{2}+G’{’}^{2}}$ .^3,4^ The yield-stress σ_Y_ is defined at the change of slope of the shear stress vs shear strain curve. Three samples were performed per sample to determine the average yield-stress under each salt concentration condition. Frequency sweep measurements (from ω = 0.1 to 100 rad/s at γ = 1%) were performed at linear viscoelastic (LVE) region to measure relative elastic (*G*’ storage modulus) and viscous (*G*” loss modulus) components. The in situ UV-induced cross-linking of the hybrid HAMA-CHIMA bulk and granular hydrogels was obtained by placing a UV-source under the glass bottom plate of the rheometer. The curing was followed by performing a time sweep experiment (ω = 1 rad/s at γ = 1%) for 600 s. Frequency sweeps were then performed on the cured samples using the same parameters as those mentioned before.

### Compressive Testing

An Anton Paar MCR302 rheometer (Anton Paar Co., The Netherlands) with a plate–plate geometry (top plate diameter 10 mm) was used to perform uniaxial compressive testing. 1.5 and 0.5 M samples were molded as cylinders (diameter, 10 mm; thickness, 4 mm) and used as prepared. To prepare 0.15 M samples, the granular hydrogel was prepared at 1.5 M NaCl, molded as cylinders, and then immersed in a 0.15 M bath for 1 day prior to measurements. The diameter of all samples was measured before the test. Then, the samples were placed on the flat and smooth stainless-steel bottom-plate of the rheometer that was maintained at 20 °C. For all of the measurements, the probe was lowered to establish contact with the hydrogels. This height is further used as the thickness of the sample (*h*_0_). Then, the compressive testing was performed at a speed of 20 μm/s. The force–displacement curve was converted into stress (σ)–strain (ε) curves using the following formulas: $\varepsilon =\frac{h_{0}-h}{h_{0}}$ and $\sigma =\frac{4F}{\pi d^{2}}$ , where *h* is the gap between the top and bottom plates. The results from the triplicate measurements were plotted and reported.

### Confocal Laser Scanning Microscopy (CLSM)

The dilute hybrid HAMA-CHIMA microgels dispersions as well as the jammed granular hydrogels prepared at 1.5, 0.5, and 0.15 M NaCl were imaged using a Leica Stellaris 8 Falcon using two different excitation laser lines and separate detectors corresponding to Rhodamine (λ_ex_ = 543 nm, detection window = 548 −624 nm) and BODIPY (λ_ex_ = 501 nm, detection window = 505–541 nm).

### Microgels Size

The microgels made from four different batches were mixed and dispersed in 1.5, 0.5, or 0.15 M NaCl aqueous solutions, and five different images were taken with a light microscope from microgels in each salt concentration. The diameter of at least 300 microgels was measured from the images using ImageJ software, and the average ± the standard deviation is reported.

### Microgels Packing Density

The packing density of the jammed granular hydrogels prepared at 0.15, 0.5, and 1.5 M NaCl was calculated by image analysis using ImageJ based on at least 5 images obtained by CLSM. The detailed protocol is as follows: (i) the CLSM image was converted to a 8-bit image leading to a gray-level type image. (ii) The image was converted to black and white using the option Threshold which can be found under the tab Image > Adjust. The background was set to dark, the microgels appeared white, and the threshold level was adjusted. These images were later analyzed by using a Python code to calculate the number of white and black pixels. The packing density value was then reported as the percentage of pixels appearing white compared to the total number of pixels within the picture. The Python code is uploaded to a public repository and can be accessed through the following link: https://github.com/ArminAmirsaedghi/PACKING_DENSITY.

### Granular Hydrogels Swelling

The granular hydrogels prepared with 1.5 M NaCl were obtained following the procedure reported above. Then, they were placed in a Plexiglas cylindrical mold (diameter = 10 mm and height = 4 mm) and sealed with two polycarbonate sheets. After this, they were carefully removed and immersed for 24 h in a 25 mL aqueous bath containing either 1.5, 0.5, or 0.15 M NaCl that was refreshed every 2 h within the first 6 h. The relative volume change was determined by taking as reference the volume of the original granular hydrogel (*V*_0_) prepared at 1.5 M NaCl before immersion, following the formula: relative volume change = [(*V*_*X*_ - *V*_0_)/*V*_0_] × 100, where *X* is the salt NaCl concentration of the immersion bath.

### Statistical Analysis

A one-way ANOVA test with Tukey posthoc test was applied to the data to determine statistical significance using GraphPad Prism 8.0. *p*-values under 0.05 were considered statistically significant (*p < 0.05, **p < 0.01, ***p < 0.001, and ****p < 0.0001).

## Results and Discussion

### HAMA-CHIMA Hybrid Microgels Synthesis

To prepare the hybrid HA-CHI microgels, we investigated the possibility of co-cross-linking HA and CHI through covalent bonding by free radical polymerization. For this, HA and CHI were first methacrylated by reaction with methacrylic anhydride following a widely reported procedure (Experimental Section and Figure S2).^33^ Hyaluronic acid methacrylate (HAMA) and chitosan methacrylate (CHIMA) were obtained with a methacrylation degree of respectively 14 mol % compared to HA repeat units and 11 mol % compared the d-glucosamine units of CHI (Figure S3). HAMA and CHIMA were further fluorescently labeled with BODIPY and Rhodamine B, respectively, to enable their visualization by confocal laser scanning microscopy (CLSM).

The microgels were then obtained by emulsifying an aqueous precursor solution containing 2.5 wt % HAMA, 2.5 wt % CHIMA, 1.5 M NaCl, and 0.1 wt % LAP in light mineral oil containing 2 wt % SPAN 80 as emulsifier (see Figure 1a). At this stage, it is important to note the two following points: (i) the NaCl concentration was chosen to be high enough to screen the electrostatic interaction between HAMA and CHIMA and avoid subsequent electrostatic complexation in the precursor solution; (ii) the concentrations of HAMA, CHIMA, and LAP were first confirmed by linear rheological experiments to be high enough to obtain a bulk hydrogel under UV irradiation (390–405 nm) (Figure S4). After complete emulsification, the system was UV-irradiated and continuously stirred for 10 min to initiate the radical polymerization of the methacrylate pendant groups of HAMA and CHIMA. Finally, the microgels were purified by centrifugation-redispersion cycles first against light mineral oil, then against 1.5 M NaCl aqueous solutions containing 2 wt % TWEEN 20, and finally against a 1.5 M NaCl aqueous solution only. More details concerning the synthesis procedure are provided in the Experimental Section.

**Figure 1:**
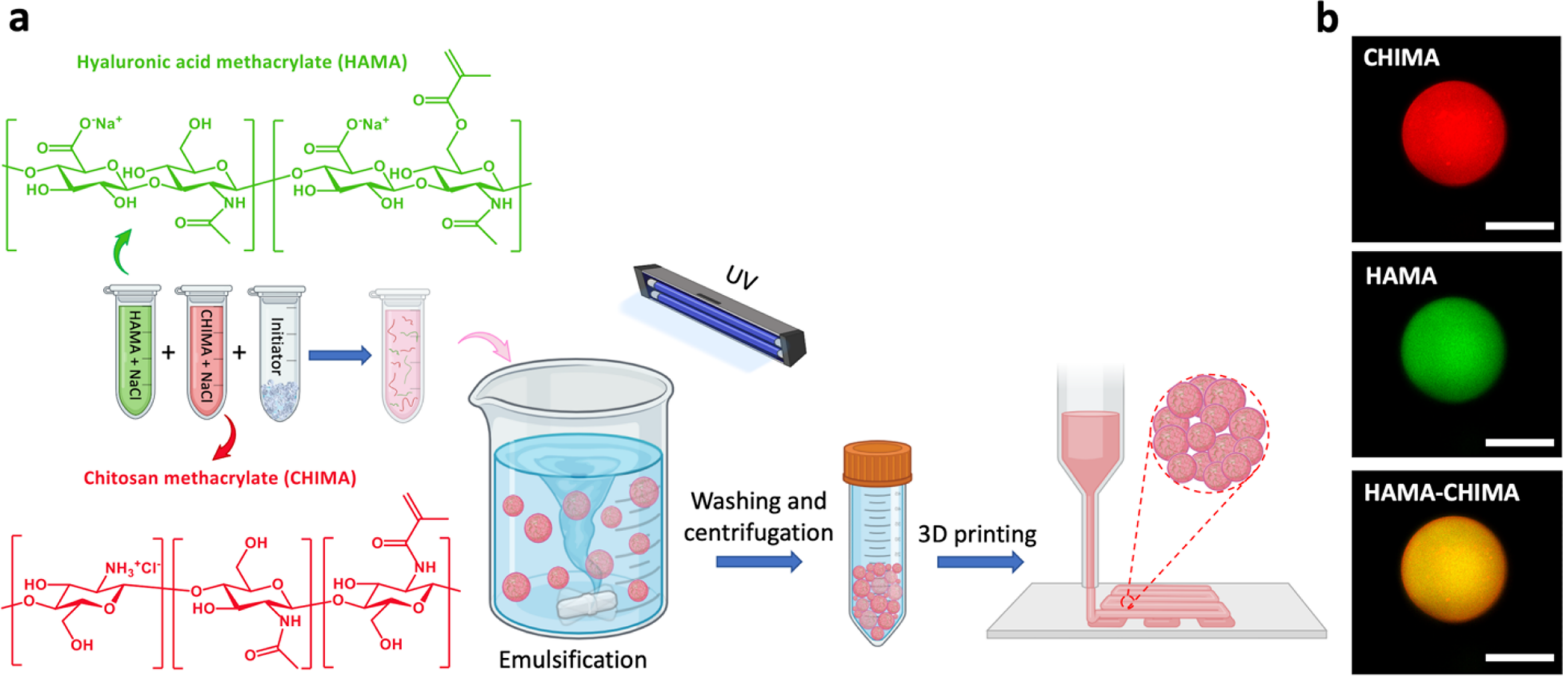
(a) Synthesis of HAMA-CHIMA hybrid microgels by UV-mediated cross-linking in an inverse batch emulsification process, followed by their jamming into granular hydrogels at 1.5, 0.5, or 0.15 M NaCl by centrifugation (4500 rcf) that were further 3D-printed. (b) CLSM pictures of a single HAMA-CHIMA hybrid microgel just after preparation, purification, and redispersion in a 1.5 M NaCl aqueous solution (same conditions as the preparation state) where only the red (top), only the green (center), or both (bottom) fluorescent channels are represented. Scale bar is 40 μm.

To assess whether or not HAMA and CHIMA were successfully co-crosslinked within single particles, we first imaged the microgels at low concentration (<1 wt %) by CLSM (Figures 1b and S5). In these pictures, we clearly see colocalized BODIPY labeled HAMA (green channel) and rhodamine B labeled CHIMA (red channel) within all microgels. In addition, the green and red fluorescence emission signals related to each of the polymers appear to be homogeneously distributed throughout the microgels, indicating no unwanted phase separation, whether it is dissociative (precipitation of one of the polymers) or associative (complex coacervation). Consequently, the UV-induced covalent co-crosslinking between HAMA and CHIMA appears to be successfully translatable from a bulk hydrogel system (Figure S4) to a dispersed emulsified state leading to the formation of hybrid microgels. To the best of our knowledge, this is the first report of the synthesis of hybrid microgels made of two oppositely charged polyelectrolytes.

As commonly reported in the literature, electrostatic associations between oppositely charged polyelectrolytes (including HA and CHI) are promoted by lowering the salt concentration of the medium.^23,25,34^ For this specific HA-CHI system (identical molecular weight ≈ 30–50 kg/mol), we already showed that complex coacervation occurs below a critical NaCl concentration that lies between 0.5 and 0.6 M and that the strength of the interactions gradually increased with decreasing salt concentration.^27^ Thus, we again used CLSM to measure the average size of the individual microgels that were first prepared and purified at 1.5 M NaCl, then redispersed into aqueous solutions containing three characteristic NaCl concentrations: (i) at 1.5 M which is above the critical concentration for chains association; (ii) at 0.5 M which is just at the limit of complexation; (iii) and at 0.15 M which is far below the critical salt concentration and similar to physiological salt concentrations. The corresponding CLSM pictures, the microgels’ size distribution determined by image analysis (see Experimental Section for further details), and light microscopy images are reported in Figures 2a,b and S6. Before further discussion, it is worth noticing that the production method of the microgels, namely batch emulsification, leads to microgels with large size distributions.^33^ At 1.5 M NaCl, the microgels exhibit an average diameter of 34 ± 15 μm. When decreasing the NaCl concentration to 0.5 M, the average diameter of the microgels remained unchanged (33 ± 14 μm) but a further decrease of NaCl concentration to 0.15 M NaCl led to a significant decrease of their average diameter (22 ± 10 μm). This decrease of almost 33% in the average diameter (70% in volume) from 1.5 M (or 0.5 M) to 0.15 M NaCl is attributed to the strengthening of the electrostatic associations between HAMA and CHIMA inside individual microgels (Figure 2c). It is interesting to note that bulk HAMA-CHIMA hydrogels showed a similar trend. Hydrogels immersed in 1.5 and 0.5 M NaCl exhibited an identical swelling, while hydrogels immersed in 0.15 M contracted significantly (Figure S7). These results prove that electrostatic interactions induced by a decrease of the salt concentration below the critical concentration for electrostatic association (CSC) is an efficient stimulus to trigger a volume change of the microgels.

**Figure 2:**
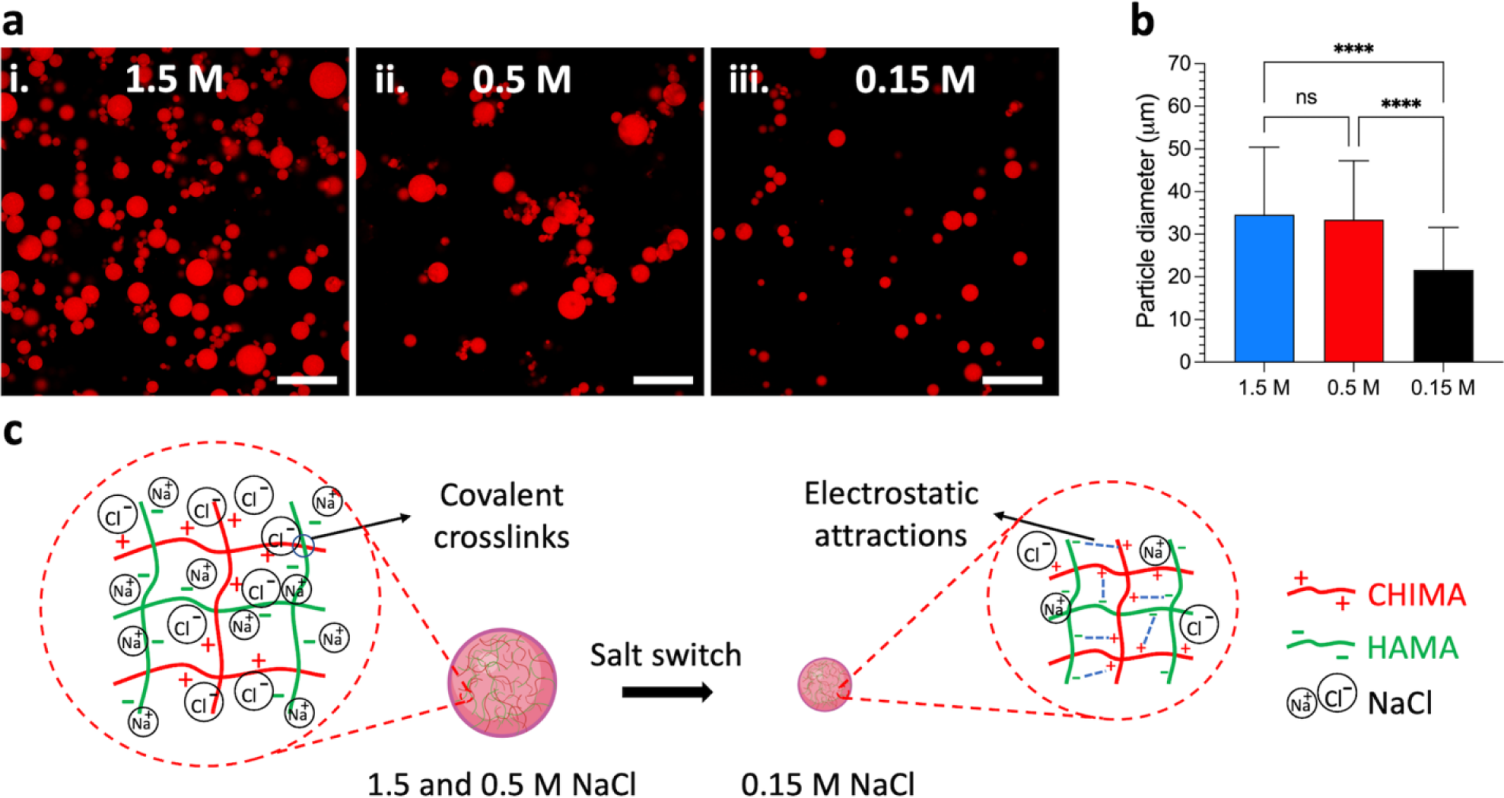
(a) Fluorescence pictures of HAMA-CHIMA hybrid microgels dispersed in aqueous solutions containing **(i)** 1.5, **(ii)** 0.5, and **(iii)** 0.15 M NaCl. Only the fluorescence signal of the red channel is reported here. Scale bar = 200 μm. (b) Average diameter of the HAMA-CHIMA hybrid microgels at 1.5, 0.5, and 0.15 M NaCl calculated from light microscopy images (Figure S6). (c) Schematic illustration of the proposed internal structural changes within the HAMA-CHIMA microgels as a function of the salt concentration. For NaCl concentrations ≥ 0.5 M NaCl, electrostatic interactions between oppositely charged polyelectrolytes within the microgels are effectively screened by salt ions. When the salt concentration is lowered to 0.15 M NaCl, reduced ionic screening allows for the formation of electrostatic associations between oppositely charged groups on the polymers, leading to a more compact microgel structure.

### Rheological Properties and Microstructure of the HAMA-CHIMA Granular Hydrogels

To produce HAMA-CHIMA hybrid granular hydrogels, the purified microgels were dispersed in aqueous solutions containing 1.5, 0.5, or 0.15 M NaCl and further jammed by centrifugation. The viscoelastic and yield-stress properties of the resulting granular hydrogels were first characterized by rheology. Figure 3a depicts the evolution of the storage (*G*’) and loss (*G*″) moduli as a function of angular frequency at low deformation (strain = 1%, well within the linear viscoelastic region) for the three hydrogels. All the hydrogels showed the typical behavior of jammed glassy microgel systems that are characterized by an angular frequency independent *G*’ value that is well above the *G*″ value.^3,4,35^ The hydrogels consequently all behaved as elastic solids that do not flow at rest. Nevertheless, a significant effect of the NaCl concentration on the stiffness of the hydrogels was still observed. First, by decreasing the concentration from 1.5 to 0.5 M NaCl, no significant difference in the plateau modulus value, *G*_p_, was observed with *G*_p,1.5/0.5_ ≈ 3 kPa. However, by decreasing the NaCl concentration further to 0.15 M, the plateau modulus value significantly increased up to *G*_p,0.15_ ≈ 20 kPa. As control, we also performed frequency sweep experiments in the low deformation regime (ω = 0.1–100 rad s^–1^, γ = 10%, well within the linear viscoelastic region) on bulk HAMA-CHIMA hydrogels (Figure S7c). Similar to the granular hydrogels, the bulk hydrogels immersed in 1.5 and 0.5 M NaCl showed almost identical *G*’ while the hydrogel immersed in 0.15 M NaCl showed a considerable increase in *G*’.

**Figure 3:**
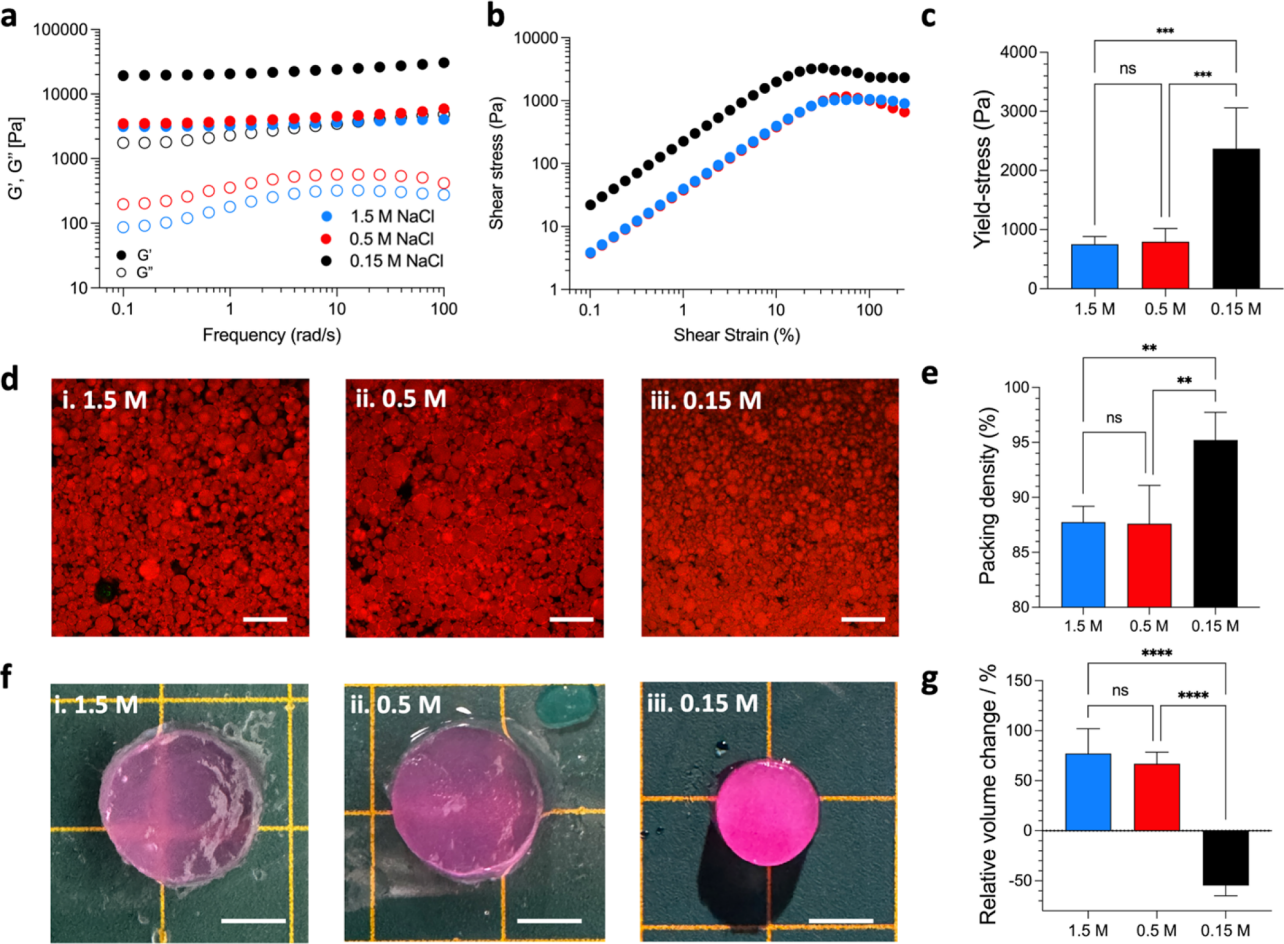
Rheological characterization of HAMA-CHIMA granular hydrogels prepared at 1.5, 0.5, and 0.15 M NaCl. (a) Frequency sweep (ω = 0.1–100 rad s^–1^, γ = 1%), (b) shear stress vs shear strain sweep (ω = 1 rad s^–1^, γ = 0.1–1000%) and (c) extracted yield-stress values. (d) CLSM pictures of the granular hydrogels prepared at (i) 1.5 M, (ii) 0.5 M, or (iii) 0.15 M NaCl. The scale bars are 200 μm. (e) Measured microgel packing density. (f) Camera pictures of the granular hydrogels 24 h after immersion in a (i) 1.5 M, (ii) 0.5 M, or (iii) 0.15 M NaCl aqueous bath. The scale bars are 5 mm. (g) Relative volume swelling change of the granular hydrogels. The hydrogel prepared at 1.5 M NaCl is taken as a reference (Figure S11).

The hydrogels’ flow properties that dictate their 3D-printability were then investigated by submitting them to increased deformation levels (from 0.1 to 1000%) at low frequency (1 rad/s) (Figure 3b). From this, the so-called yield-stress of the hydrogels could be extracted (Figures 3c and S8) that is defined as the stress where the onset of flow is observed. Following the trend observed in the linear rheological properties, we can determine that the yield-stress, hallmarking the initiation of flow, of the hydrogel containing 0.5 M NaCl remained unchanged compared to the hydrogel prepared at 1.5 M NaCl (σ_Y,0.5/1.5_ ≈ 750–800 Pa). Then, when the NaCl concentration was decreased to 0.15 M, it increased significantly up to σ_Y,0.15_ ≈ 2400 Pa. Both the increase in *G*_p_ and σ_Y_ are a clear signature of the increase in strength of the electrostatic associations between HAMA and CHIMA. The increase of both *G*_p_ and σ_Y_ by lowering the NaCl concentration to 0.15 M lies in the same order of magnitude that was observed for similar systems composed of linear polyelectrolyte chains.^26,27^ Additional uniaxial compression measurements confirmed the marked increase of mechanical strength of the granular hydrogel immersed in a 0.15 M NaCl bath compared to hydrogels prepared either at 1.5 or 0.5 M NaCl (Figure S9). Consequently, polyelectrolyte complexation proves itself to be as efficient for this granular system compared to systems composed only of linear chains to enhance its cohesiveness and self-supporting properties.

The rheological and mechanical properties of granular hydrogels are extremely dependent on their microstructure and, more precisely, on the microgel packing density also known as the volume fraction of microgels. We then used CLSM to image the granular hydrogels prepared at NaCl concentrations of 1.5, 0.5, or 0.15 M and further determined the microgels packing density by image analysis (Figures 3d,e and S10). The 2D images reported are obtained at different *X*-*Y* areas inside the hydrogels to obtain an average packing density of the microgels. More details about the image analysis procedure can be found in the Experimental Section. First, we can observe that the packing density remained constant by decreasing the salt concentration from 1.5 M (packing density = 87.8 ± 1.4%) to 0.5 M (packing density = 87.6 ± 3.5%) (Figure 3e). At 0.15 M, the microgel packing density increased significantly to reach 95.2 ± 2.5%.

Figure 3f represents the pictures of the hydrogels after 24 h immersion in a **(i)** 1.5, **(ii)** 0.5, and **(iii)** 0.15 M NaCl aqueous bath (the picture of the as-prepared hydrogel is reported in Figure S11). We measured the relative volume change of these hydrogels compared to the volume of the hydrogel prepared at 1.5 M and reported it in Figure 3e. First, the hydrogels immersed in 1.5 and 0.5 M NaCl baths exhibited a prominent swelling of 77.3 ± 24.6% and 67 ± 11.6% after 24 h, respectively. Oppositely, by lowering the NaCl concentration to 0.15 M, a macroscopic contraction of the granular hydrogel (−54.7 ± 10.3%) was evidenced. Strikingly, the hydrogel immersed in 0.15 M NaCl was completely opaque. This opacification is commonly observed for polyelectrolyte complexes that undergo a “salt switch” due to the formation of segregated water-rich and polymer-rich domains that increase the light scattering of the system.^26,36,37^ Consistent with the observed granular hydrogels’ swelling profiles, TGA measurements confirmed that the polymer content remained nearly identical (4–5 wt %) in the granular hydrogels prepared at 1.5 and 0.5 M samples, while a considerable increase in polymer content (>20 wt %) was revealed in the granular hydrogel desalted in a 0.15 M NaCl aqueous bath (Figure S12).

Altogether, these results highlight that an increase in the strength of the electrostatic complexation between HAMA and CHIMA at 0.15 M NaCl not only induces a deswelling of the single microgels but also reinforces the electrostatic interactions between neighboring microgels that lead to the formation of a more compact hydrogel. At higher NaCl concentrations, the poor connectivity due to inexistent or weak electrostatic associations between polymer chains within the microgels and between microgels leads to a lower packing density. We hypothesize that this can also hamper the integrity of the hydrogels when immersed for a prolonged time in an aqueous medium. By its intrinsic design, our study nevertheless does not allow for the decorrelation of the effects of packing density and interparticle cross-linking on the final mechanical properties of the hydrogels. They are indeed both interdependent and are dictated by the strength of electrostatic associations, whether they occur between chains inside the same microgels or in between neighboring microgels. Yet, qualitative insights can be gained by comparing the rheological properties of the granular hydrogels prepared at 1.5 M NaCl, with and without post-UV cross-linking performed after mixing with 0.1 wt % LAP photoinitiator (Figure S13). In this case, the packing density of the microgels is, at first glance, supposed to remain constant, while UV irradiation introduces covalent interparticle cross-links. Although the nature of covalent bonds differs fundamentally from that of electrostatic interactions, this comparison still provides a useful indication of the potential reinforcing effect of increased interparticle connectivity. The plateau modulus increased by a 5-fold (from ≈ 2000 to 10,000 Pa) indicating the significant impact of covalent interparticle cross-linking on the stiffness of the resulting granular hydrogel. This relative increase lies in the same order of magnitude as other granular hydrogel systems reinforced by interparticle covalent bonds interactions.^38–40^ When it comes to reinforcement of interparticle interactions by electrostatic association without affecting the packing density, the work of the De Angelis et al. appear to be the most relevant.^41^ The authors observed that blending negatively charged poly­(acrylic acid), pAA, microgels with poly­(diallyldimethylammonium chloride), pDADMAc, linear chains enabled to increase the plateau modulus of their granular hydrogel by 3.5 fold independently of the molecular weight of the pDADMAc chains (<100 to 450 kg/mol). They assigned this reinforcement to the bridging between neighboring microgels by pDADMAc chains that enable to efficiently withstand higher loads.

Overall, the tuning of the salt concentration appears to be a relevant strategy to enhance the cohesion and structural integrity of 3D-printed HAMA-CHIMA granular hydrogels in aqueous media, without requiring postprinting cross-linking for scaffold shape retention.

### 3D Printing of HAMA-CHIMA Granular Hydrogels Scaffolds

The 3D extrusion printing of the HAMA-CHIMA granular hydrogel prepared at 1.5 M NaCl into a square-mesh grid scaffold was attempted. Figure 4a,b shows that it was possible to print 2- and 4-layer scaffolds with a strand-to-strand distance of 2 mm in air without observing any shape relaxation. Further, it was possible to print a 2-layer scaffold with an even shorter strand-to-strand distance of 1.2 mm without observing any merging of the strands, indicating the possibility to target structures with variable resolutions (Figure 4c-i,ii). Scaffolds made from granular hydrogel inks prepared at 0.5 M NaCl were also successfully printed, showing a similar level of accuracy as the scaffolds obtained from the inks prepared at 1.5 M NaCl (Figure S14). This high level of printability that is reflected by the calculated high Pr values (Pr_1.5_ = 1.017 ± 0.026 and Pr_0.5_ = 0.971 ± 0.002) is afforded by their relatively high yield-stress value (σ_Y,0.5/1.5_ ≈ 750–800 Pa) that prevents the collapse or flow of the printed strands. Yet, it is worth noting that it was impossible to 3D print continuous scaffolds from the granular hydrogel prepared at 0.15 M NaCl within a reasonable range of air pressure, as the hydrogel was too cohesive (σ_Y,0.15_ ≈ 2400 Pa). This observation indicates that there exists an upper limit of yield-stress above which 3D printing becomes impossible, that is, in the precise case of the hydrogel prepared at 0.15 M, due to excessive interparticle electrostatic associations.

**Figure 4:**
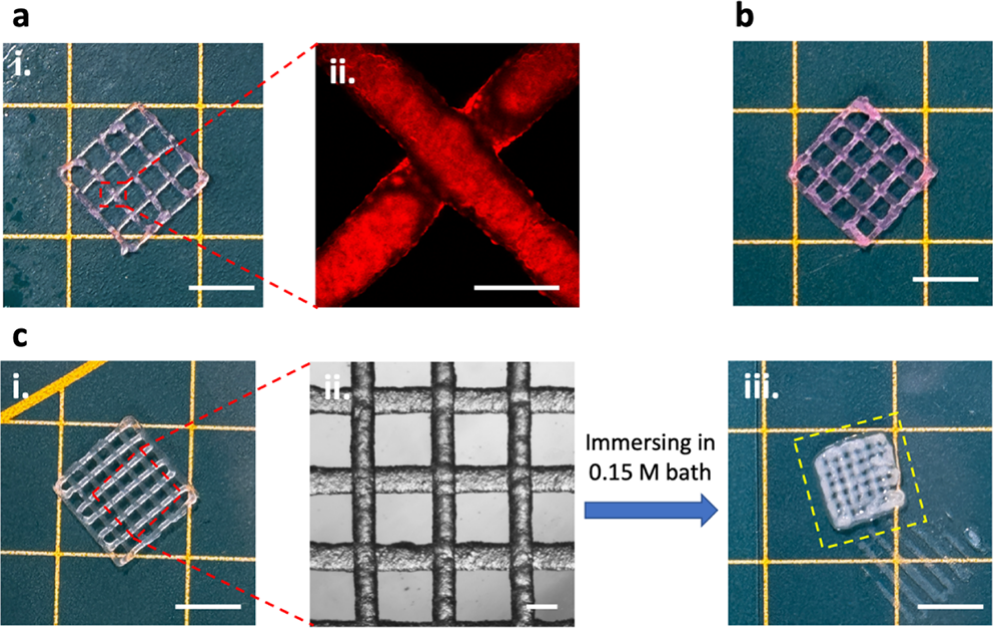
(a) (i) Camera picture of a 2-layer 3D-printed scaffold of HAMA-CHIMA RB granular hydrogel ink prepared at 1.5 M NaCl (strand-to-strand distance = 2 mm) and (ii) its corresponding CLSM image taken from one of the *X*-*Y* junctions. (b) Camera picture of a 4-layer 3D-printed scaffold of HAMA-CHIMA RB granular hydrogel ink prepared at 1.5 M NaCl (strand-to-strand distance = 2 mm). (c) (i) Camera picture of a 2-layer 3D-printed scaffold of HAMA-CHIMA granular hydrogel ink prepared at 1.5 M NaCl (strand-to-strand distance = 1.2 mm) and (ii) its corresponding light microscopy image. (iii) Camera picture of the scaffold after 1 h immersion in a 0.15 M NaCl aqueous bath (the yellow dashed area shows the dimension of the printed structure before immersion). The scale bars are 5 mm for the camera pictures, 200 μm for the CLSM image, and 500 μm for the light microscopy image.

The structural integrity in aqueous medium of the 2-layer scaffolds printed in air was then investigated by immersing them in either 1.5 or 0.15 M NaCl baths. Scaffolds immersed in a 1.5 M NaCl bath disassembled after mild agitation, reflecting the lack of interparticle interactions at a high ionic strength (Figure S15). In contrast, scaffolds immersed in a 0.15 M NaCl bath contracted and showed increased structural integrity (Figures 4c-iii and S16). Moreover, similar to the results obtained for the granular hydrogel cylinders, we could observe an evident contraction of around 30 vol % of the printed scaffold accompanied by its opacification after immersion in a 0.15 M NaCl bath. This deswelling, due to the enhancement of the electrostatic interactions, can improve the structural integrity of the printed scaffolds in a similar manner as that was already reported by Gong et al.^19^ After equilibrium was reached, the scaffolds remained intact even under mild agitation, demonstrating that salt-responsive electrostatic interactions can significantly enhance scaffold mechanical stability (Movie S1).

## Conclusion

In the present study, we combined in a unique system the intrinsic salt-responsive properties of polyelectrolyte complexes and yield-stress behavior of granular hydrogels to develop 3D-printable hydrogels inks with tunable mechanical properties and enhanced structural integrity in aqueous media, without requiring postprinting cross-linking for scaffold shape retention. We first synthesized negatively charged hyaluronic acid methacrylate (HAMA) and positively charged chitosan methacrylate (CHIMA) and co-cross-linked them into a single hybrid microgel under UV in an inverse batch emulsion. The microgels prepared at 1.5 M NaCl, a concentration well above the critical salt concentration for electrostatic association (CSC) between HAMA and CHIMA, showed an average diameter of around 34 μm with homogeneous incorporation of both HAMA and CHIMA as confirmed by confocal laser scanning microscopy. By decreasing the salt concentration to 0.15 M NaCl, well below the CSC, a clear contraction of the microgels, showing an average diameter of around 22 μm, was measured due to the increase of the strength of the electrostatic association between the polyelectrolytes. The microgels were further jammed by centrifugation. The rheological properties of the resulting granular hydrogels showed both an increase in their plateau modulus and yield-stress upon decreasing the NaCl concentration. The hydrogels also showed concomitantly a dramatic contraction of up to 70% in volume that is comparable to the extent of contraction observed for the single microgels. Finally, we successfully printed scaffolds from the hybrid granular hydrogel prepared with 1.5 M NaCl that could exhibit a high shape fidelity. By immersing these scaffolds in a bath containing 0.15 M NaCl, we could prevent the dispersion of the microgels in the medium without the need of any postprinting cross-linking process. In the meantime, due to the strong electrostatic association intra- and intermicrogels the scaffolds shrunk by 30%. Thus, by the design of hybrid polyelectrolyte microgels, the local deswelling due to electrostatic complexation between HAMA and CHIMA could be successfully translated to the whole scaffold. This self-stabilizing mechanism distinguishes our strategy from prior methods that rely heavily on external chemical cross-linkers to maintain printed architectures. Moreover, the resulting hydrogel scaffolds exhibit salt-responsive contraction behavior, adding a dynamic and reversible dimension to the material that is scarcely addressed in the current granular hydrogel literature. Such responsiveness is particularly relevant in the context of tissue engineering, where mimicking the biomechanical adaptability of native tissues is highly desirable. Importantly, the dynamic contraction capacity of the printed scaffolds holds strong potential for enhancing the regenerative performance of muscle tissue mimics, which require not only structural fidelity but also mechanical functionality to support cellular growth and alignment. As such, this work offers a significant advancement in the design of smart, self-assembling granular hydrogels that combine printability, stability, and responsiveness—key criteria for next-generation biofabrication platforms.

## Supplementary Material





## Notes

This paper contains Supporting Information. Setup for UV polymerization of HAM-CHIMA microgels. Reaction schemes and ^1^H NMR of HAMA and CHIMA. CSC of the HAMA-CHIMA system. Rheological characterization of the bulk HAMA-CHIMA hydrogel. CLSM pictures of the dilute HAMA-CHIMA microgels. Swelling degree of the HAMA-CHIMA bulk hydrogels. Shear stress vs shear strain and CLSM pictures of the granular HAMA-CHIMA hydrogels (DOCX)
Mechanical agitation of the 2-layers square mesh scaffold printed with the HAMA-CHIMA granular hydrogel ink prepared at 1.5 M NaCl and immersed in a 0.15 M NaCl aqueous bath (MOV)
