# Supplementary material for: 3D-Printable Granular Hydrogel Composed of Hyaluronic Acid-Chitosan Hybrid Polyelectrolyte Complex Microgels
Source: Biomacromolecules. 2025 May 22;26(6):3641–50. doi: 10.1021/acs.biomac.5c00228 (PMC12152954; doi:10.1021/acs.biomac.5c00228)
Supplement: Supplementary file 1 [file bm5c00228_si_001.docx]

**Supporting Information for**

**3D-Printable Granular Hydrogel Composed of Hyaluronic Acid-Chitosan Hybrid Polyelectrolyte Complex Microgels**

Armin Amirsadeghi ^a^, Shahriar Mahdavi ^a^, Paula Jager ^a^, Marleen Kamperman ^a^, Julien Es Sayed ^b,c*^

^a^ Zernike Institute for Advanced Materials, University of Groningen, Nijenborgh 3, 9747 AG Groningen, the Netherlands.

^b^ Biotechnology Centre, The Silesian University of Technology, B. Krzywoustego 8, 44-100, Gliwice, Poland

^c^ Department of Biomedical Engineering, University of Groningen, University Medical Center Groningen, A. Deusinglaan 1, Groningen, AV 9713, The Netherlands

E-mail: [Julien.EsSayed@polsl.pl](mailto:Julien.EsSayed@polsl.pl), [j.s.es.sayed@rug.nl](mailto:j.s.es.sayed@rug.nl)

**
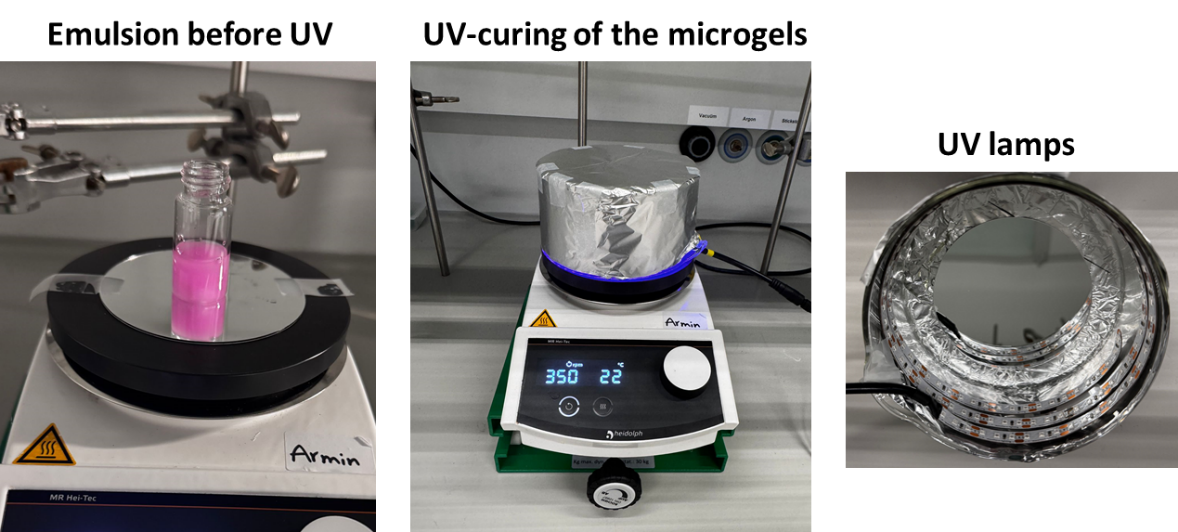
**

**Figure S1.** Setup used for the UV-induced crosslinking of the HAMA-CHIMA hybrid microgels.

**
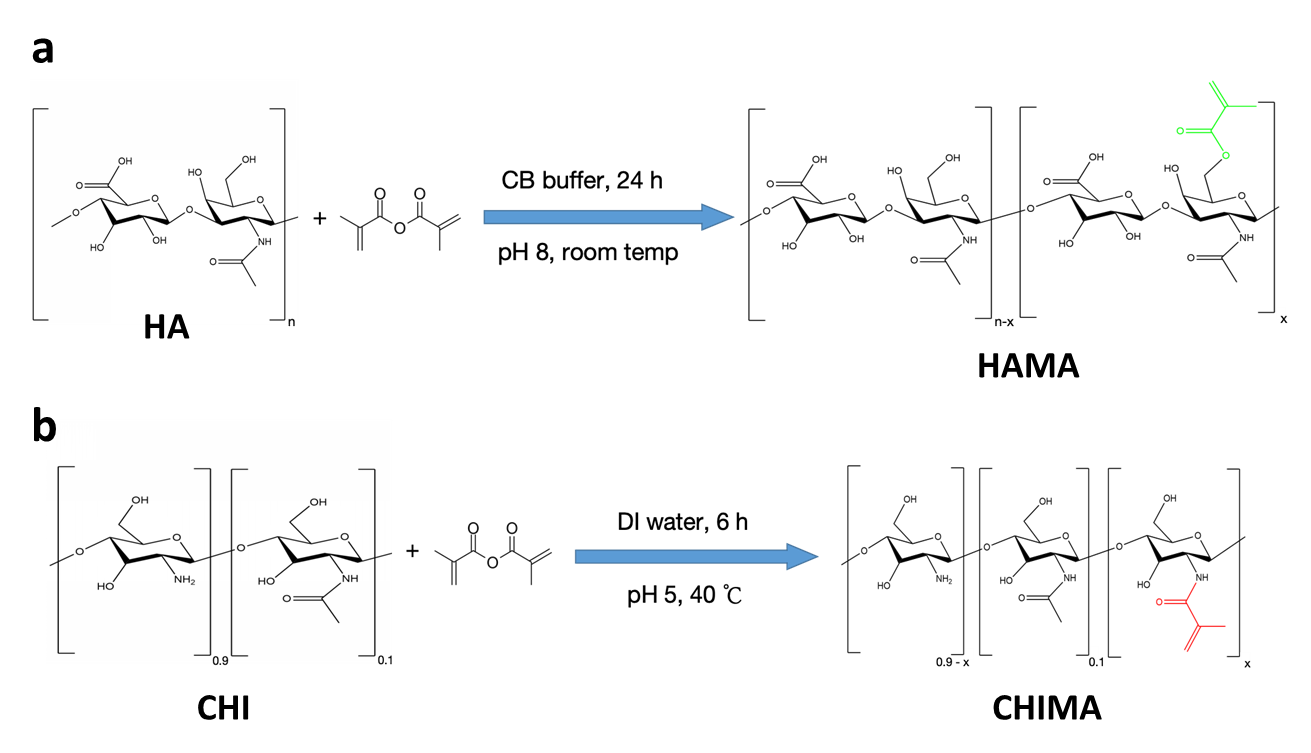
**

**Figure S2.** Reaction scheme for the synthesis of HAMA and CHIMA.


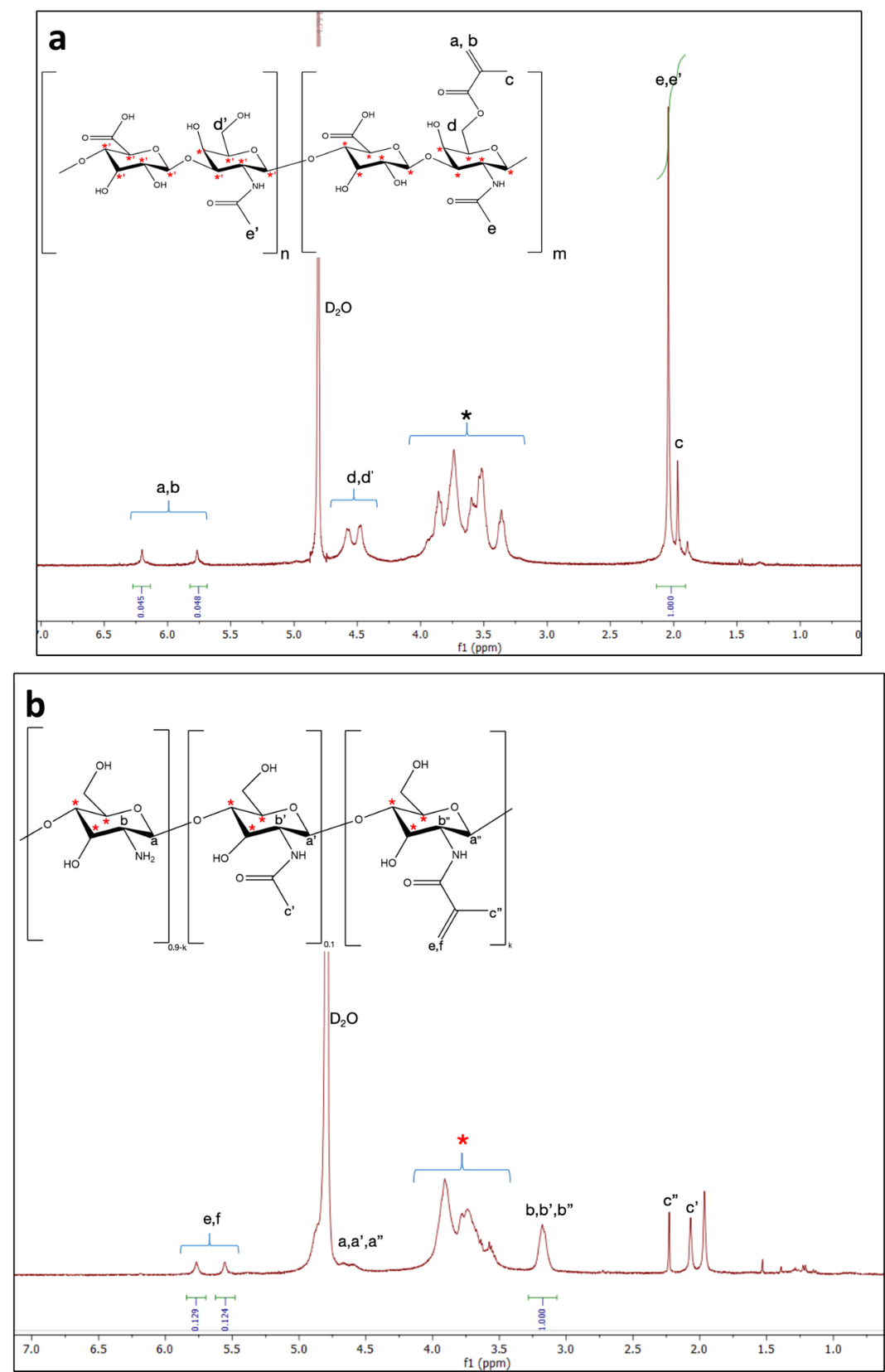


**Figure S3.** ^1^H NMR spectrum (D_2_O) of **(a)** HAMA and **(b)** CHIMA. The degree of methacrylation was determined using the following formula (1) for HAMA and the formula (2) for CHIMA. For HAMA it was determined to 14 mol% and for CHIMA to 11 mol%.

Formula for the calculation of the DM for HAMA and CHIMA:


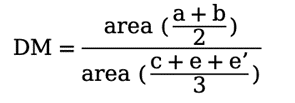


**(1)**


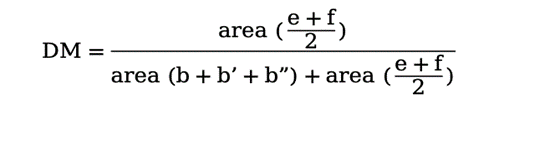


**(2)**

**
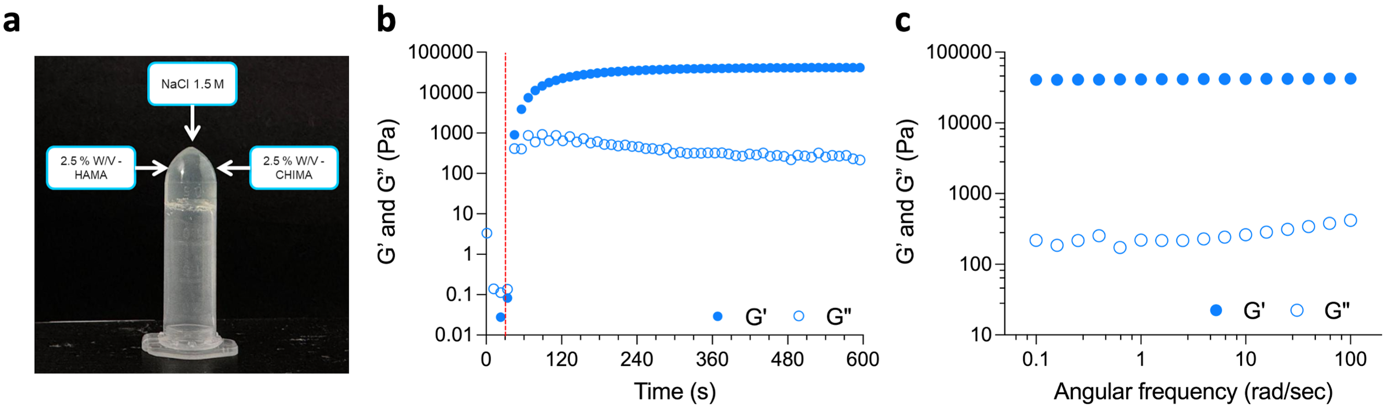
**

**Figure S4.** Rheological characterization of the HAMA-CHIMA bulk hydrogel prepared at 1.5 M NaCl. **(a)** Picture of the hydrogel after UV curing in an inverted tube, **(b)** time sweep measurement (ω = 1 rad s^−1^, γ = 1%) under UV curing (the red dashed-line showing the time in which the UV lamp turned on), and **(c)** frequency sweep after 10 minutes UV curing (ω = 0.1 - 100 rad s^−1^, γ = 1%). Both visual observation and rheology confirm the complete gelation of the HAMA-CHIMA stock solution containing 2.5 wt.% HAMA, 2.5 wt.% CHIMA, 1.5 M NaCl and 0.1 wt.% LAP.


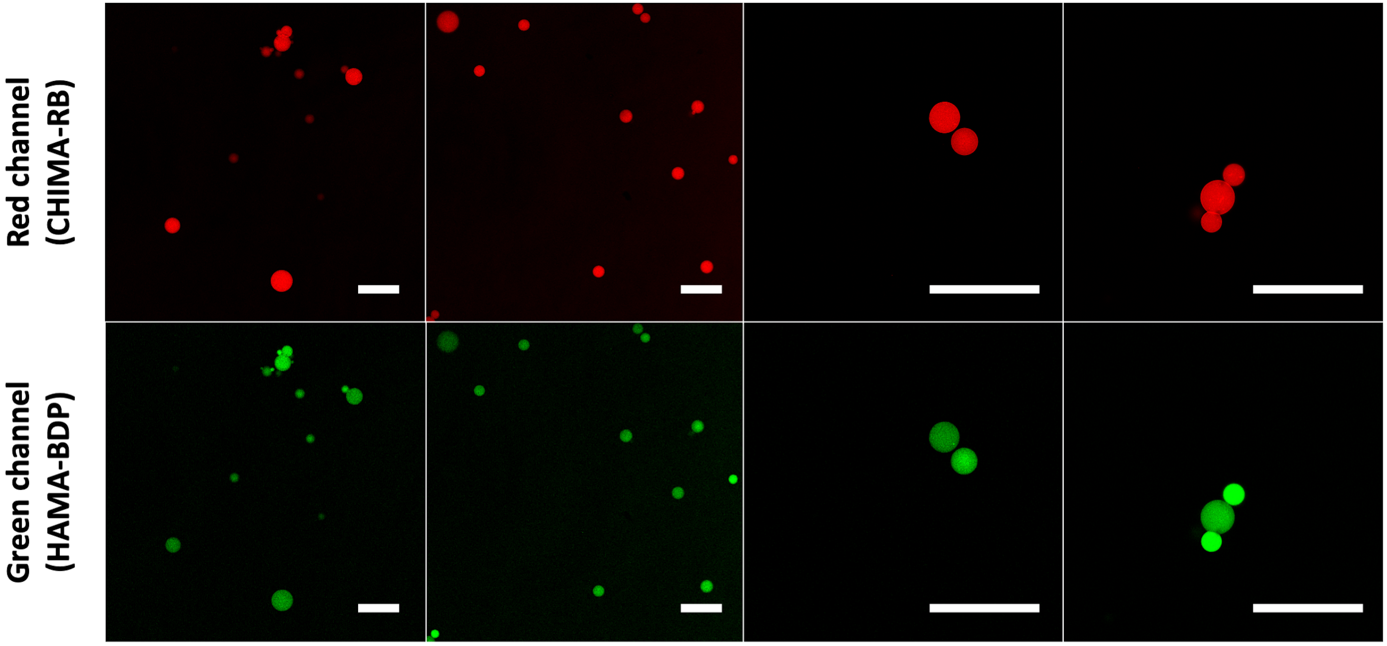


**Figure S5.** CLSM images of the HAMA BDP-CHIMA RB microgels prepared at 1.5 M NaCl at dilute state. The scale bars are 200 µm.

**
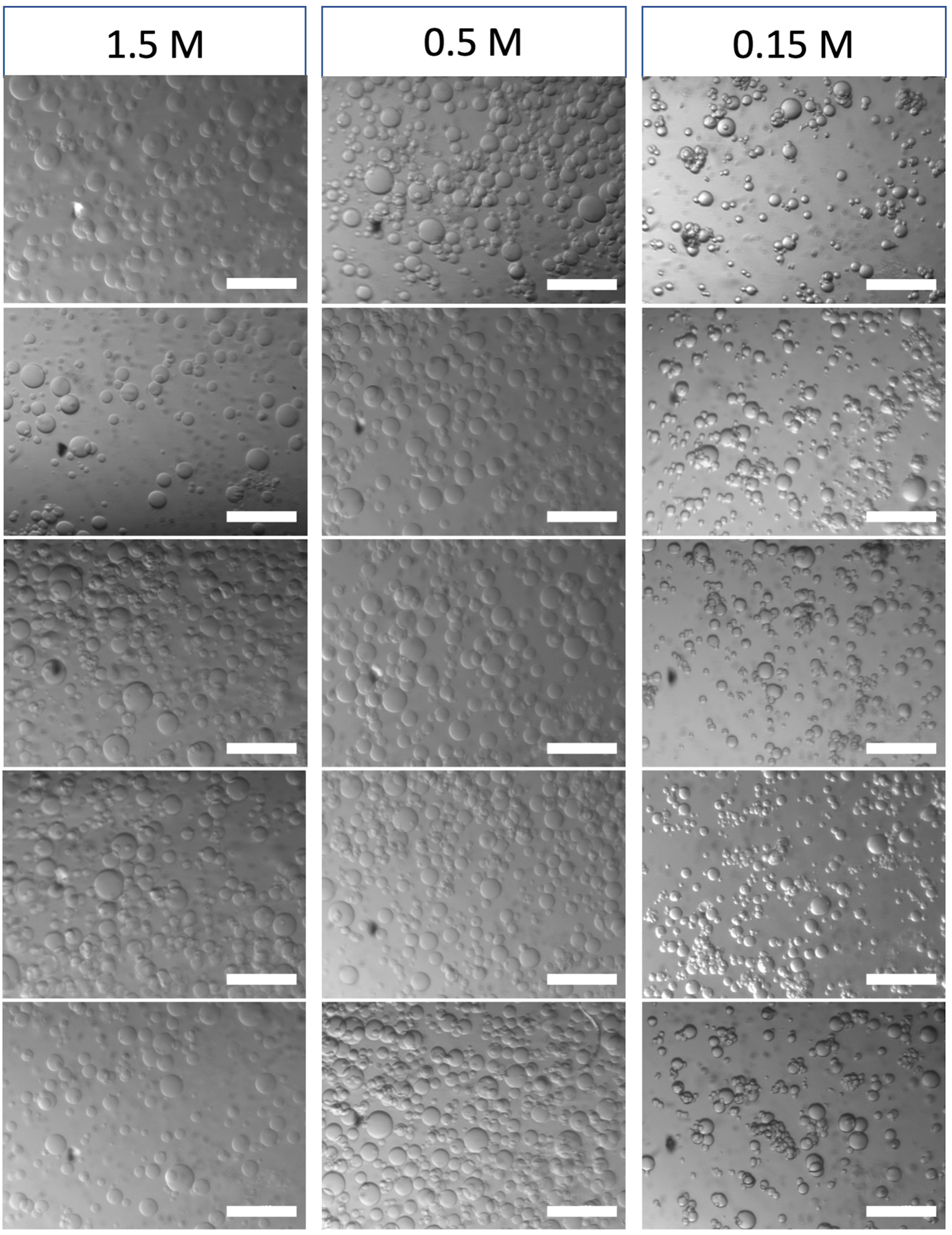
**

**Figure S6.** Light microscopy images of the HAMA-CHIMA microgels dispersed in aqueous solutions containing 1.5, 0.5, and 0.15 M NaCl. The scale bars are 200 µm.


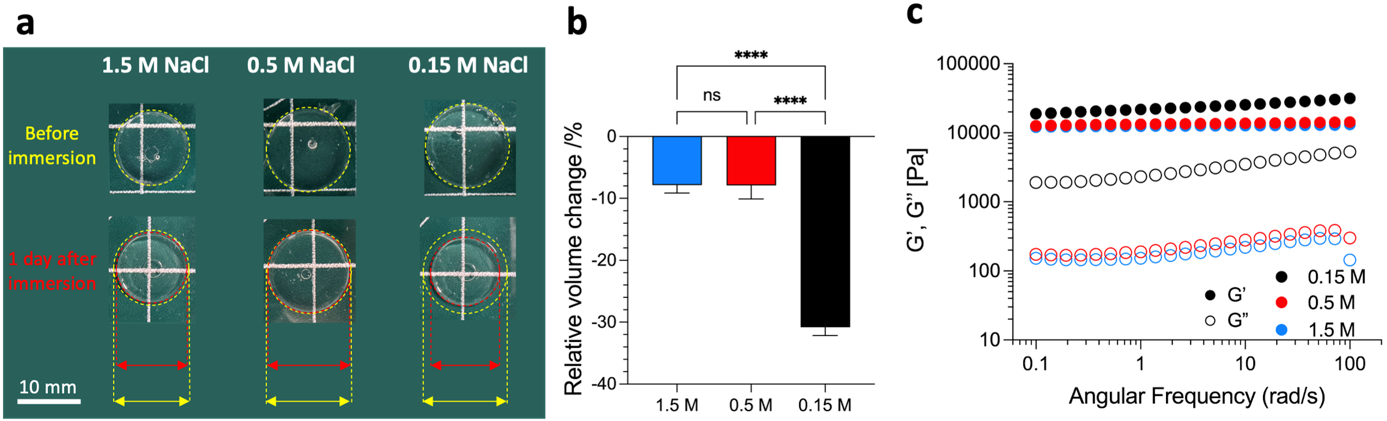


**Figure S7.** **(a)** Pictures, **(b)** relative volume change, and **(c)** frequency sweep measurements of bulk HAMA-CHIMA hydrogels after 1-day immersion in 1.5, 0.5, and 0.15 M NaCl aqueous solutions.


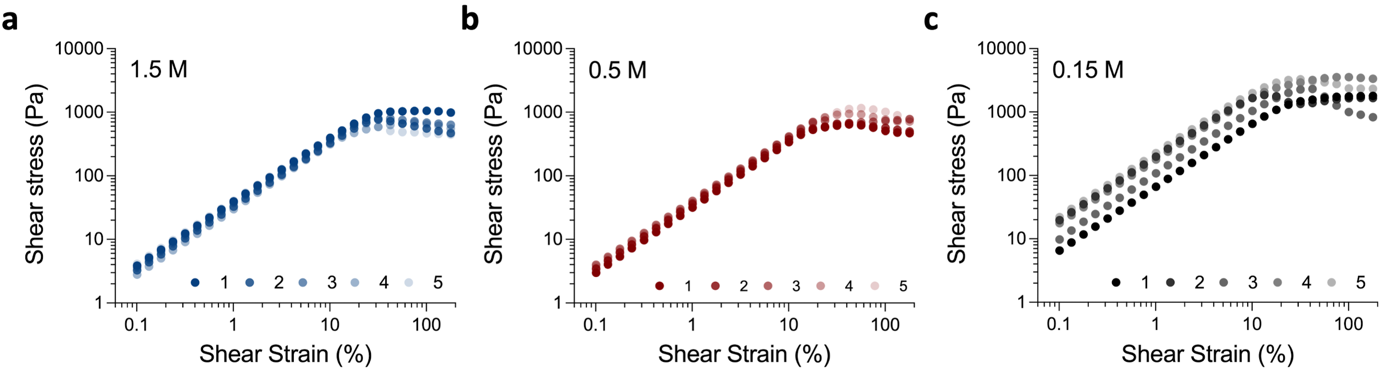


**Figure S8.** Shear stress (σ) vs shear strain curve of the HAMA-CHIMA granular hydrogels prepared in **(a)** 1.5, **(b)** 0.5, and **(c)** 0.15 M NaCl aqueous solutions. The shear stress is calculated using the following formula: $\sigma=\gamma*\sqrt{{G'}^{2}+{G''}^{2}}$. The yield stress σ_Y_ is defined at the change of slope of the shear stress vs shear strain curve.


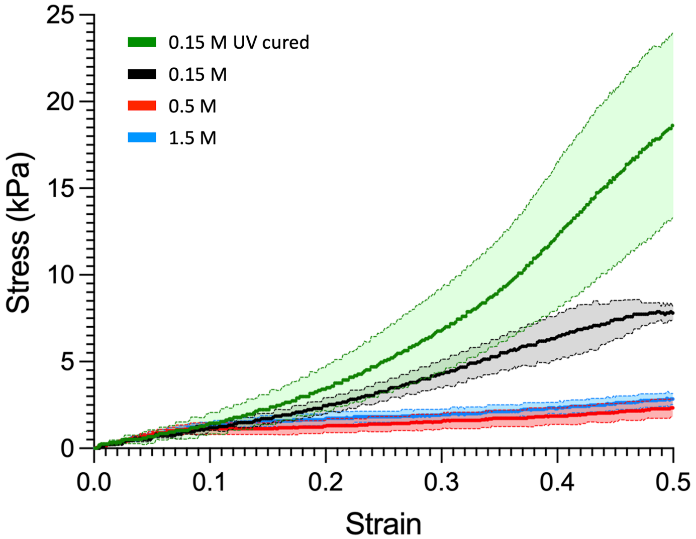


**Figure S9.** Stress-strain curves obtained by uniaxial compression testing on the granular hydrogels prepared at 1.5 M, 0.5 M, 0.15 M NaCl and on the 1.5 M granular hydrogel exposed to UV (in presence of 0.1 wt.% LAP) and desalted in a 0.15 M NaCl solution. The curves are an average of three measurements. The standard deviation is represented by a shaded area around the curves.


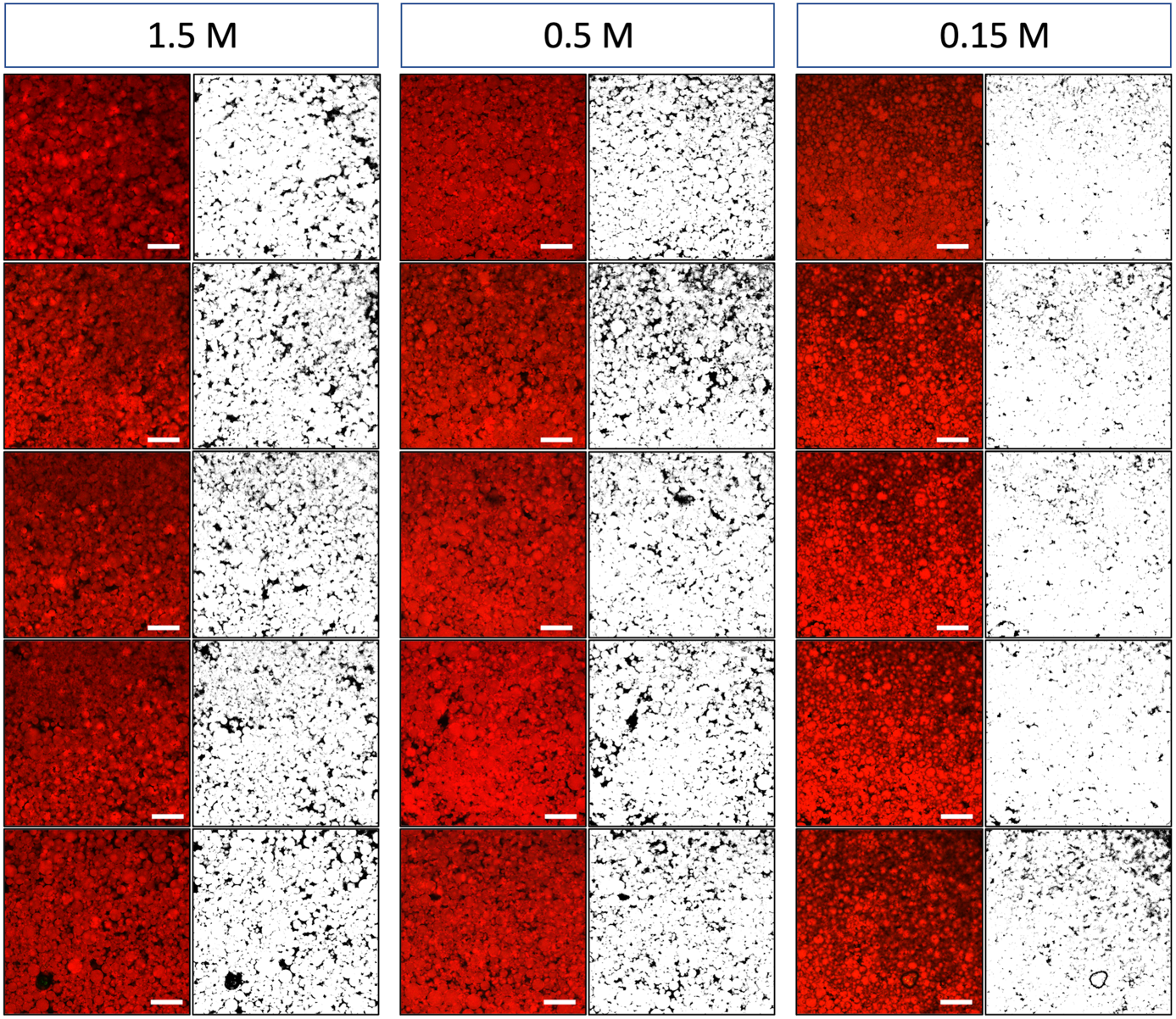


**Figure S10.** CLSM pictures of the granular hydrogels prepared at 1.5, 0.5, and 0.15 M NaCl. The scale bars are 200 µm. The black and white image analysis obtained through ImageJ analysis that allows to determine the microgels packing density are represented aside of each picture.


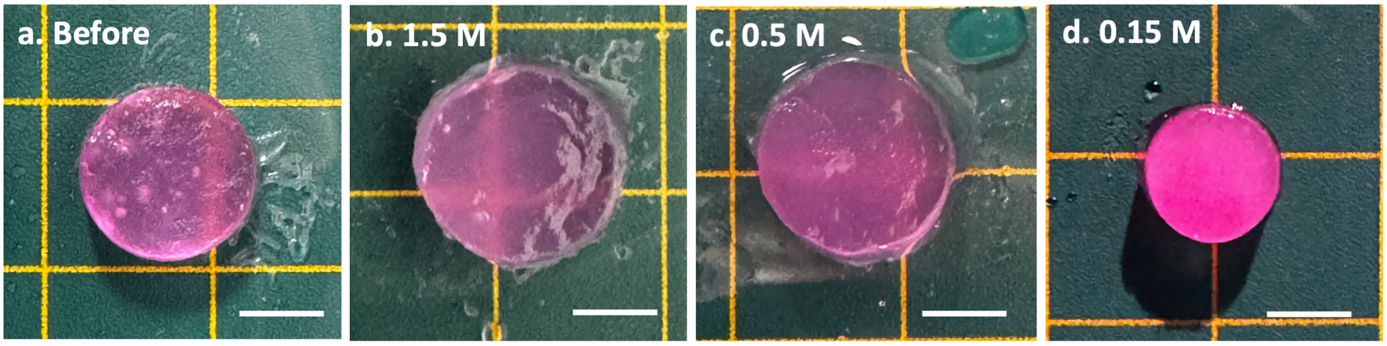


**Figure S11.** Camera pictures of the granular hydrogel **(a)** prepared at 1.5 M NaCl before immersion and 24h after immersion in a **(b)** 1.5, **(c)** 0.5, and **(d)** 0.15 M NaCl aqueous bath. The scale bars are 5 mm.


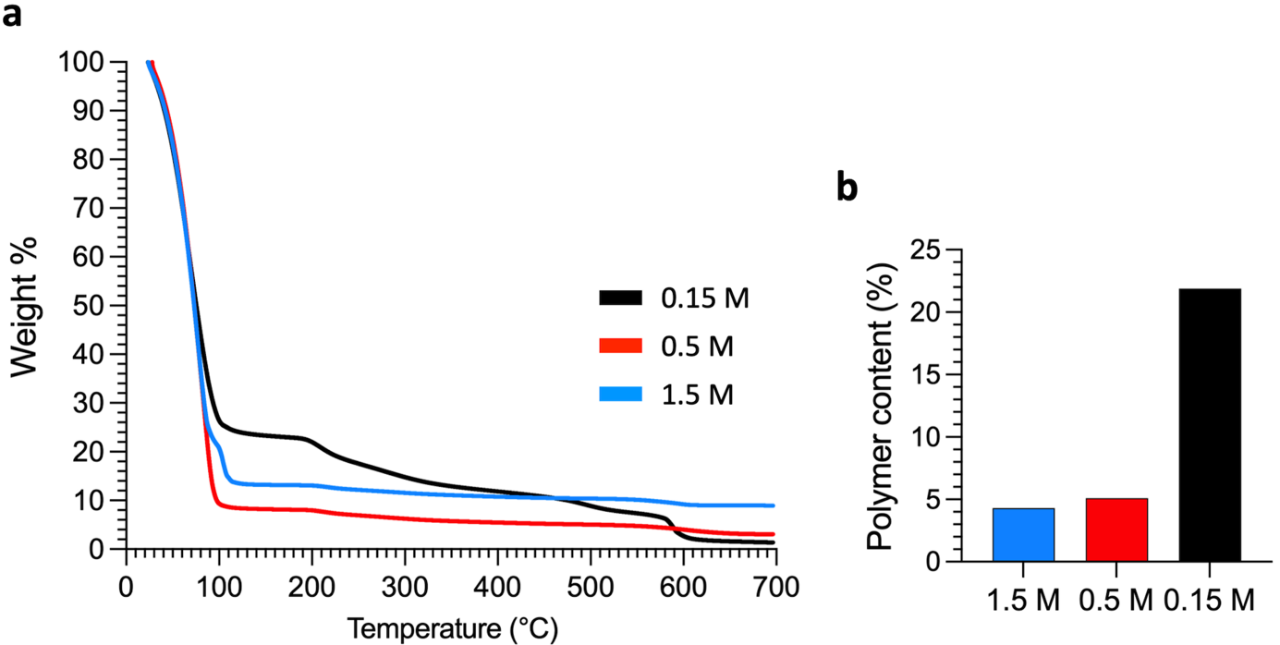


**Figure S12. (a)** Thermograms obtained from TGA experiments and **(b)** derived polymer content of the granular hydrogels prepared at 1.5 M, 0.5 M and 0.15 M NaCl.


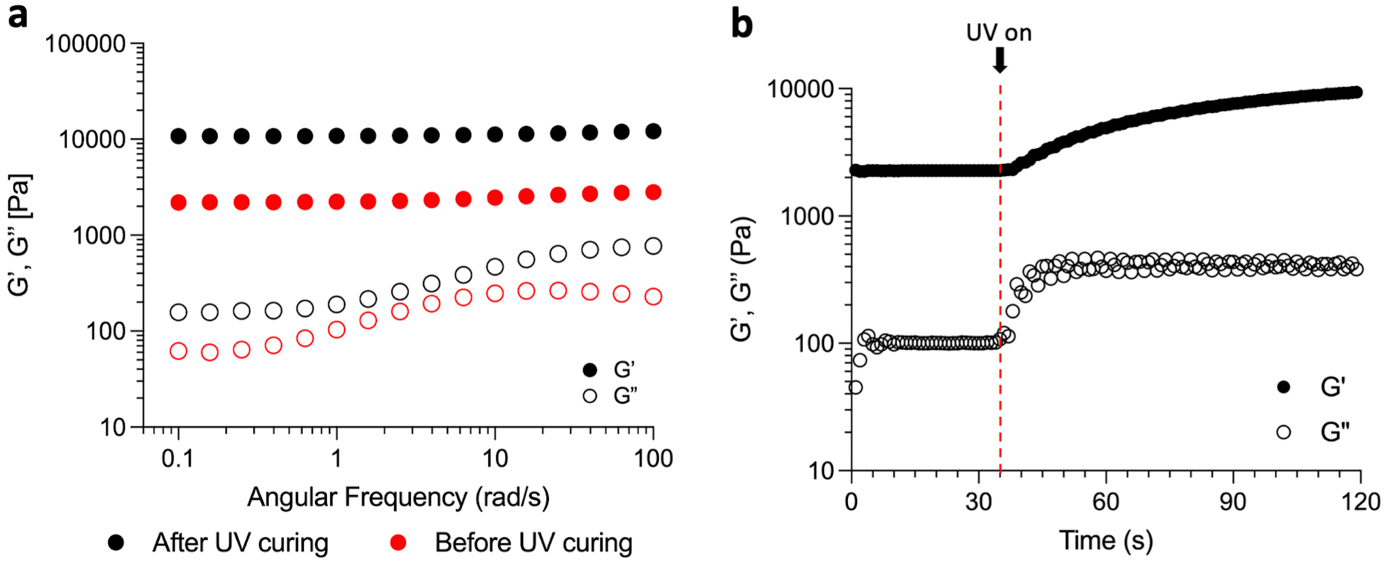


**Figure S13.** Rheological characterization of the HAMA-CHIMA granular hydrogel prepared at 1.5 M. **(a)** Frequency sweep before and after UV curing (ω = 0.1 - 100 rad s^−1^, γ = 1%). **(b)** Time sweep (ω = 1 rad s^−1^, γ = 1%) during UV curing. The red dashed line shows the time that UV lamp was turned on.


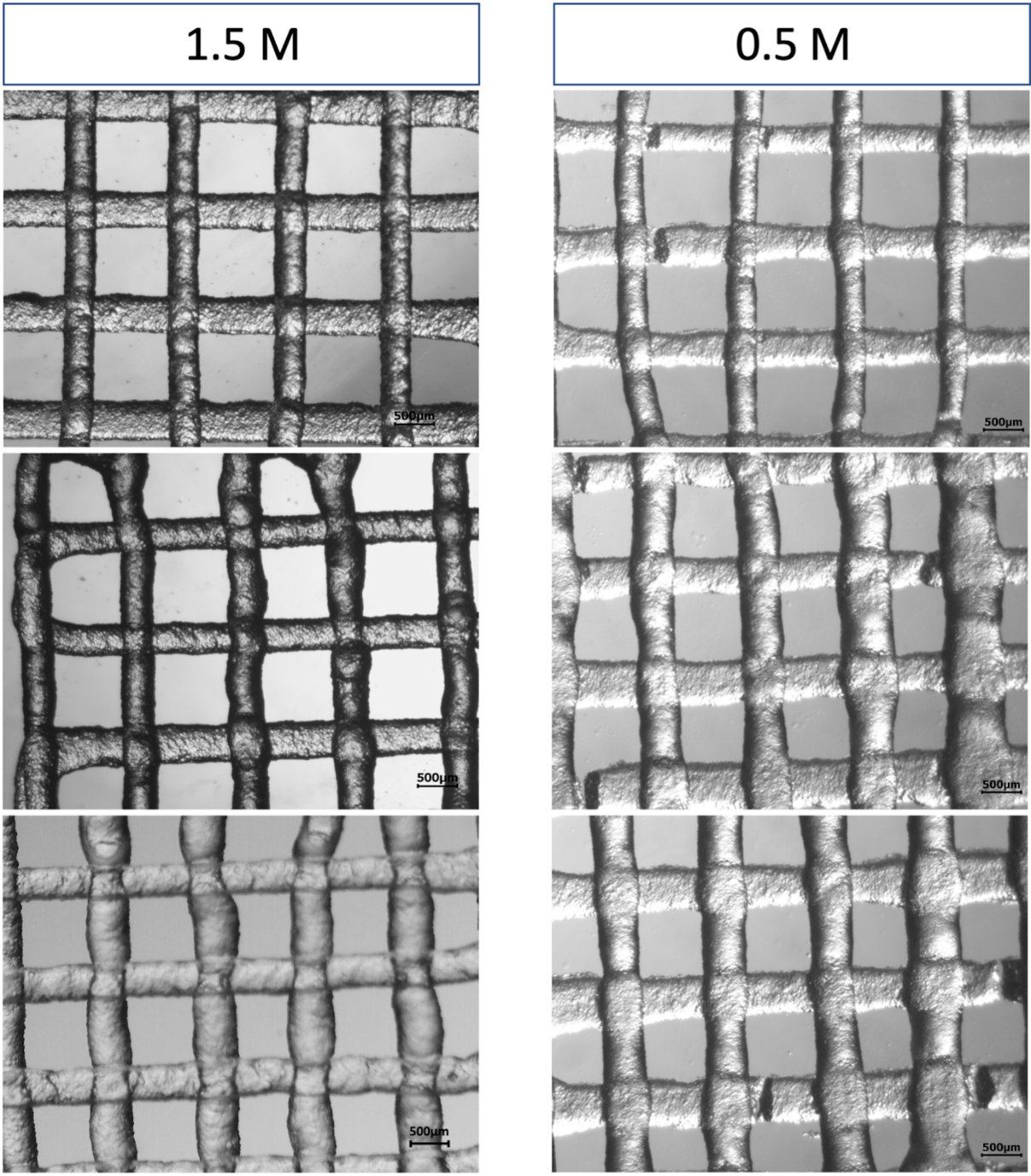


**Figure S14.** Light microscopy images of two-layer square-mesh scaffolds printed using HAMA–CHIMA granular hydrogel inks prepared at 1.5 and 0.5 M NaCl. Each panel shows three representative prints per condition to illustrate printing consistency. The strand-to-strand distance was set to 1.2 mm. These images were also used to calculate the printability index (Pr) for each formulation, as reported in the main text.

**
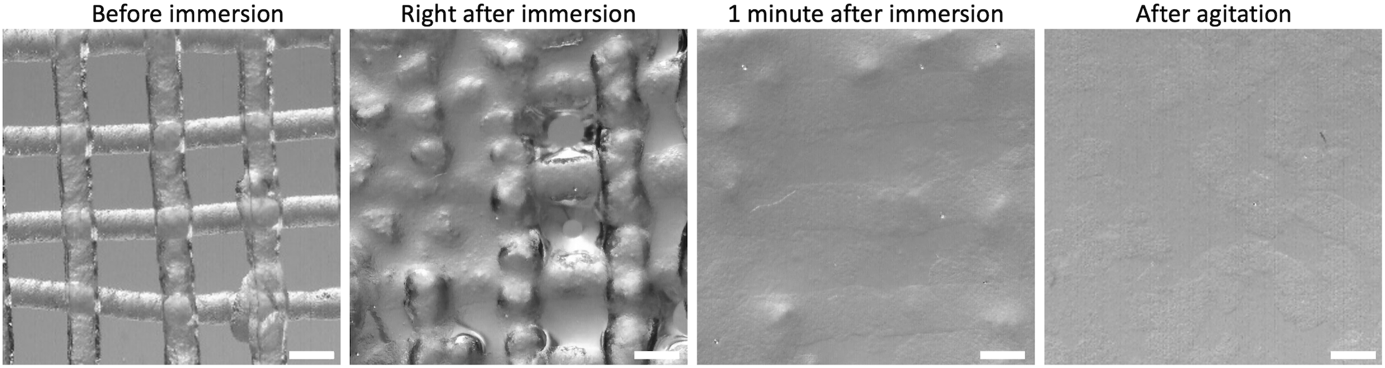
Figure S15.** Microscopy images showing the structural integrity of a 3D-printed two-layer square-mesh scaffold (8 × 8 mm², 1.2 mm strand-to-strand distance) prepared using HAMA–CHIMA granular hydrogel inks at 1.5 upon immersion in 1.5 M NaCl bath at different states after immersion. Scale bars are 500 µm.


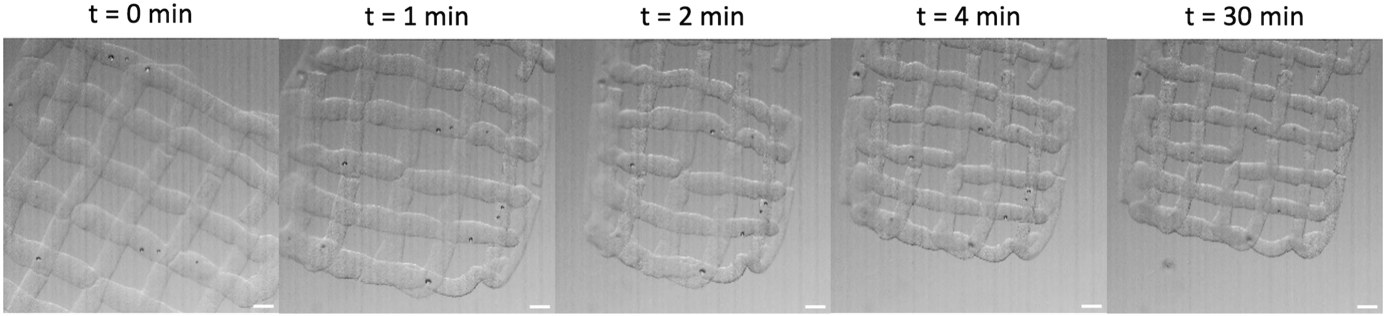


**Figure S16.** Time-lapse microscopy images showing the salt-responsive behavior of a 3D-printed two-layer square-mesh scaffold (8 × 8 mm², 1.2 mm strand-to-strand distance) prepared using HAMA–CHIMA granular hydrogel inks at 1.5 M NaCl upon immersion in 0.15 M NaCl bath. Scale bars are 500 µm.

**Movie S1.** Mechanical agitation of the 2-layers square mesh scaffold printed with the HAMA-CHIMA granular hydrogel ink prepared at 1.5 M NaCl and immersed in a 0.15 M NaCl aqueous bath.
